# Allicin regulates Treg/Th17 balance in mice with collagen‐induced arthritis by increasing the expression of MEKK2 protein

**DOI:** 10.1002/fsn3.2034

**Published:** 2021-04-07

**Authors:** Yuling Zhang, Yufang Gong

**Affiliations:** ^1^ Department of Rheumatism and Immunity Weifang People's Hospital Weifang City China

**Keywords:** collagen‐induced arthritis model, MEKK2, rheumatoid arthritis, Treg/Th17 balance

## Abstract

To study the role of Allicin in regulating Treg/Th17 ratio in splenic lymphocyte by increasing the expression of MEKK2 protein in MAPK signaling pathway, and to explore the mechanism of immune response in mice with collagen‐induced arthritis (CIA). Mouse CIA model was induced by chicken collagen type II, and experimental mice were randomly divided into NC group, Model group, and Allicin group. HE staining was used to compare the degree of joint pathological damage in mice of each group, and Masson staining to observe the proliferation of collagen tissue in each group. Flow cytometry detected Treg/Th17 ratio in splenic lymphocytes. Furthermore, RT‐PCR and WB were used to detect the mRNA and protein expression of related transcription factors and inflammatory factors Foxp3, ROR‐γt, and IL‐17A, as well as MEK2 protein expression in splenic lymphocytes. The results showed that Allicin treatment could reduce the severity of arthritis and the proliferation of collagen fibers on the surface of cartilage and bone joints in CIA mice. Compared with NC group, Treg decreased and Th17 increased in spleen lymphocyte of Model group (*p* < .01); after Allicin treatment, Treg increased while Th17 decreased significantly (*p* < .01). Meanwhile, MEKK2 protein expression in spleen lymphocyte of Model group decreased compared to that in NC group (*p* < .01), and MEK2 protein expression increased significantly after Allicin treatment (*p* < .01). To sum up, the present study suggests that MEKK2 protein plays an important role in the pathogenesis of CIA model. In terms of mechanism, Allicin may play a therapeutic role in rheumatoid arthritis (RA) by increasing the expression of MEKK2 protein and affecting Treg/Th17 ratio.

## Graphical abstract

In this work, we clearly discuss allicin had effects to improve collagen‐induced arthritis by regulation Treg/Th17 balance.

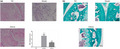

## 1 INTRODUCTION

Rheumatoid arthritis (RA) is a chronic systemic autoimmune disease featured by synovitis. The pathogenesis of RA is intricate, and its pathogenesis and progression involve a variety of immunological factors (Niu et al., 2012; Scott et al., 2010). T‐cell dysfunction plays an essential role in the occurrence and development of RA. The imbalance between Th1/Th2 and Treg/Th17 may be the most direct and important factor in the pathogenesis of RA.

Mitogen‐activated protein kinase (MAPK) signaling pathway is critical in the pathogenesis of RA. Its activation can regulate the secretion of pro‐inflammatory and anti‐inflammatory factors, and thus mediate the progress of RA and other autoimmune diseases (Kesavan et al., 2004). MAPK/ERK kinase kinase 2 (MEKK2) is a member of the MAP3K family in the MAPK signaling pathway, which is involved in regulating the activity of MAPK. MEKK2 and its downstream signaling pathway are significantly involved in the development and activation of T cells, and participate in the regulation of T‐cell activation and homeostasis (Hammaker et al., 2004). Prior evidence has shown that the expression level of MEKK2 in peripheral blood mononuclear cells (PBMC) of RA patients is lower than that of normal population (Hammaker et al., 2007).

Allicin is an active ingredient of garlic that possesses a wide range of pharmacological properties including antioxidant, anti‐inflammatory, neuroprotective, antibacterial, and antitumor effects. Allicin can reduce the level of nitric oxide and show anti‐inflammatory effect by reducing the levels of serum interleukin 1β (IL‐1β) and TNF‐α. At the same time, it can inhibit the expression of NF‐kB protein or nuclear translocation (Zeng et al., 2016). However, it remains unclear with respect to the effect and mechanism of Allicin in improving RA. The aim of this study is to explore whether Allicin can affect the Treg/Th17 ratio of splenic lymphocyte by regulating the key protein of MEKK2 and related transcription factors of MAPK signaling pathway via establishing collagen‐induced arthritis (CIA) model, thus playing an active role in the treatment of RA.

## 2 MATERIALS AND METHODS

### 2.1 Experimental reagent

Allicin Injection was purchased from Shanghai Hefeng Pharmaceutical Co., Ltd.

### 2.2 Experimental animals

A total of 27 male SPF DBA/I mice, aged 8 weeks, with body mass of (25 ± 3) g, were provided by Shanghai SLAC Laboratory Animal Co., Ltd.

### 2.3 Major reagents

Chicken collagen type II (Chondrex), Freund's complete adjuvant (Sigma‐Aldrich), Freund's incomplete adjuvant (Sigma‐Aldrich), FBS (Gibco), RPMI 1640 (Gibco), erythrocyte lysate (Beyotime), Masson staining solution (NanJing JianCheng Bioengineering Institute), PrimeScript™ RT Maser Mix, SYBR Premix Ex Taq™ (TaKaRa), TRIzol Reagent (Invitrogen), Pierce™ BCA Protein Assay Kit (Thermo Scientific™), anti‐MEKK2, Foxp3, ROR‐γt, IL‐17A and GAPDH (Abcam), blocking buffer of SuperBlock™ T20 (PBS), Blocking Buffer (Thermo Scientific), prestained protein Marker (Thermo), secondary antibody (HRP‐Goat‐Rabbit Jackson ImmunoResearch), antimouse CD3‐FITC, antimouse CD4‐PerCP‐cy5.5 and antimouse IL‐17‐A‐APC (eBioscience), and Cell stimulation Cocktail 500× (eBioscience).

### 2.4 Induction of CIA model

Chicken collagen type II was dissolved in 0.05 mol/ml glacial acetic acid at a concentration of 3 mg/ml and stirred overnight at 4°C. The first modeling was realized by mixing dissolved collagen type II with Freund's complete adjuvant at a ratio of 1:1 and putting it on ice. Aspiration and emulsification were performed by using emulsifying needle, and the complete emulsification was defined as indiffusion of the solution by 500 times of suction to nonproliferation in water. A subcutaneous injection of 100 μl was initiated at a distance of 2 cm from the tail root of the mice, and a transparent skin mound was made for the success of the injection. On the 21st day of the first modeling, the second immunization was performed. Collagen type II of 3 mg/ml was mixed with Freund's incomplete adjuvant in a ratio of 1:1 and emulsified completely. The injection method was the same as before with an injection volume of 50 μl, avoiding the last injection site. The arthritis index was scored every 5 days in mice of each group for 6 weeks.

### 2.5 Grouping and administration

Mice were randomly divided into normal group (NC group, nine mice), CIA model group (model group, 19 mice), and Allicin group (nine mice). Allicin was given to mice in Allicin group 1 week after the second modeling, of which the drug at a dose of 5 mg/kg was dissolved in 0.2% hydroxypropyl methyl + cellulose (HRMC) for gavage administration in Allicin group. Mice in the normal group and CIA model group were intragastrically administered with the same amount of solvent. The treatment lasted for 1 month.

### 2.6 Arthritis scoring in mice

The scoring criteria were described as follows: Normal toe, 0 point; edema in 2 small toes or ankle joint, 1 point; all toe joint edema in one claw or edema in two toe joints and ankle joint, 2 points; edema in all toe joints and soles or edema in the part below the ankle joint, 3 points; total claw edema to joint deformity, 4 points. The arthritis score (AS) of each mouse was the average score of 4 toes, with the highest score of 4. The score greater than 1 indicated that the model was successful.

### 2.7 Pathological staining of mouse joints

The knee joints of both lower limbs were fixed with 10% formalin, degreased with ethanol, decalcified with formic acid, embedded with paraffin and sectioned, followed by HE staining and Mason staining. After that, the pathological changes of synovium were observed by OLYMPUS light microscope and photographed. HE staining pathological results were assessed and calculated to evaluate the severity degree by 0–3 integral grade (O'Valle et al., 2015): 0 = normal joints; 1 = mild synovial hyperplasia with focal inflammatory cell infiltration; 2 = synovial thickening, loose and disordered arrangement, a large number of inflammatory cells infiltration, increased neovascularization, accompanied by a small amount of destruction of articular cartilage; and 3 = obvious synovial hyperplasia, pannus formation, and severe damage of articular cartilage and bone tissues. Masson staining was carried out according to the steps in the kit, in which the collagen fiber was blue and the cancellous bone was red.

### 2.8 Isolation of splenic lymphocytes

The spleen tissues of mice were taken aseptically (placed on ice). The spleen was placed in a sterile dish, then cut it into small pieces with slide, followed by grinding. The operation was performed on a culture dish containing RPMI 1640 (serum‐free) medium, and the cell mass was dispersed with a pipette after grinding. The gauze filter membrane was placed on a 15 ml tube with tweezers, and the grinding solution was absorbed and filtered on the gauze. Centrifugation was then carried out for 5 min at 1250 g/min, and cells were dispersed after a rapid discarding of the supernatant. A 2 ml erythrocyte lysate was added and shaken gently for about 1 min. When white flocculent (cell membrane) appeared gradually, the RPMI 1640 culture medium was added to stop the reaction, followed by centrifugation for 5 min at 1250 g/min resuspension and counting.

### 2.9 Detection of Treg/Th17 by flow cytometry

The cell density was adjusted to 1 × 10^6^ cells/ml; 500 μl cell suspension was added to each well of the 24‐well plate. Then, 500 μl RPMI 1640 culture medium was added to the stimulating well of IL‐17 containing 4 μl cell stimulation Cocktail per ml, RPMI 1640 culture medium was added to the nonstimulating well, and then mixed in CO_2_ incubator for 6 hr, once every 2 hr. Cells were collected and added to the flow cytometry tube, washed with precooled PBS, and then suspended in 100 μl FACS solution. Afterward, the surface flow cytometry antibody was added and incubated at 4°C for 30 min in dark, followed by the addition of 3 ml FACS solution, centrifugation at a speed of 300 *g* at 4°C for 5 min, and discarding of the supernatant. The 250 μl BD membrane breaking fixative was added and incubated at 4°C for 1 hr, followed by the addition of 3 ml FACS solution for washing and the discarding of the supernatant after centrifugation, the addition of 700 μl BD Washing Buffer for another washing and the discarding of the supernatant after centrifugation. Subsequently, membrane penetrating liquid (2 ml/tube) was added, followed by centrifugation and discarding of the supernatant, dryness of the orifice via suction, and the above process was repeat once. The suspension of cells was performed with the addition of 100 μl BD Washing Buffer, and then the supplementation of intracellular staining antibody. The cells were incubated at 4°C for 1 hr in the absence of light, with one oscillation in the process. In the final step, the cells were washed and centrifuged, followed by the discarding of the supernatant, addition of 250 μl FACS solution to resuspend cells, flow cytometer detection, and FlowJo software analysis.

### 2.10 Lymphocyte proteolysis and western blotting detection

Every 1 × 10^7^ mice spleen lymphocyte were treated with 200 μl RIPA cell lysate containing protease inhibitor. After 30 min on ice, the supernatant was centrifuged at 1300 g/min and the supernatant was absorbed. BCA method was used to quantify protein. After measuring the protein concentration, the protein was quantified at 8 μg/10 μl. The protein was denatured completely at 100°C for 10 min. Proteins were separated by SDS‐PAGE electrophoresis and then transferred to PVDF blotting membrane. With blocking buffer for sealing, the primary antibody was added and incubated at 4°C overnight; then, the secondary antibody was added and incubated at room temperature for 1 hr after membrane washing, followed by enhanced chemiluminescence (ECL), development, photographic fixing, and scanning. Image J software was used to analyze the gray value of target electrophoretic bands.

### 2.11 Total RNA extraction from lymphocytes and RT‐q PCR detection

Firstly, 1 ml cell suspension (4 × 10^6^ cells) was lysed by 1 ml TRizol, followed by the addition of chloroform and isopropanol, centrifugation, and supernatant discarding. Then, the absolute ethanol was used for rinsing and precipitation, and 30 μl DEPC solution was added to dissolve RNA after dryness. The concentration of RNA was detected by bio‐spectrophotometer. The RNA was quantified by collecting 500 ng/100 μl samples. The reverse transcription was performed at 37°C for 10 min and at 85°C for 5 s. Fluorescent PCR was performed with the kit. The template of the primer sample was 2 μl, and the total reaction system was 20 μl. The target gene was detected by 7900 RT‐q PCR using Applied Biosystems. Primer sequences (Table 1) were designed by Primer 5 software, and primers were synthesized by Sangon Biotech (Shanghai) Co., Ltd. Relative gene expression multiple of each group and normal group was quantified by subtracting the corresponding GAPDH mean value from the target gene CT value, and dividing of the results of each group divided by the normal group value at the same time, and the relative gene expression multiples between each group and the normal group were calculated. The relative quantification of gene expression was achieved by 2^−ΔΔCt^ method.

**TABLE 1:** RT‐qPCR Primer sequences

Gene name	Primer sequences
GAPDH	F: 5′‐ACTTTGGCATTGTGGAAG‐3′
	R: 5′‐GGATGCAGGGATGATGTTCT‐3′
Foxp3	F: 5′‐GAAAGAGCACATTCCAGAGTTC‐3′
	R: 5′‐ATGGCCCAGCCGATGAG‐3′
ROR‐γt	F: 5′‐CCGCTGAGAGGGCTTCAC‐3′
	R: 5′‐TGCAGGAGTAGGCCACATACA‐3′
IL‐17A	F: 5′‐CTCCAGAAGGCCCTCAGACTAC‐3′
	R: 5′‐AGCTTTCCCTCCGCATTGACACAG‐3′

### 2.12 Statistical analysis

Data were processed by SPSS 20.0 statistical software. Quantitative data were expressed by Mean ± *SD*. One‐way analysis of variance was used for intergroup comparison, and *t* test was used for pairwise comparison. The difference was statistically significant with *p* < .05.

## 3 RESULTS

### 3.1 Effect of Allicin on general conditions and AS scores of feet in mice

**FIGURE 1:**
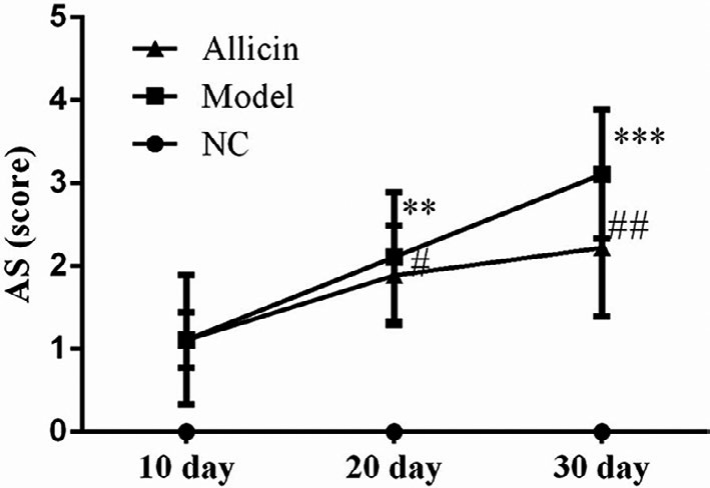
Allicin affects trend of joint integral in mice arthritis model. **: *p* < .01, ***: *p* < .001, compared with NC group; #: *p* < .05, ##: *p* < .01, compared with Model group

In the experiment, it was found that the mice in NC group had normal diet, uniform growth of body weight and normal activity. The mice in Model group had fewer diet, decreased body weight, and reduced activity. On the fifth day after the second immunization, mice showed paw swelling, joint swelling, and deformity gradually. The degree of pathological changes was aggravated, and body weight was significantly decreased. The arthritis lesion reached its peak 40 days after immunization, and AS score was significantly higher than that of mice in NC group (*p* < .01). Mice in Allicin group began to use drugs 1 week after the second modeling. Mice in the Allicin group showed better mental state than that of Model group, accompanied by decreased body weight and gradual decrease of AS score, showing significant difference between Allicin group and Model group (*p* < .01). The results suggested that Allicin treatment can reduce the severity of arthritis in mice as shown in Figure 1.

### 3.2 Effect of Allicin on pathological changes of joint tissue in mice

**FIGURE 2:**
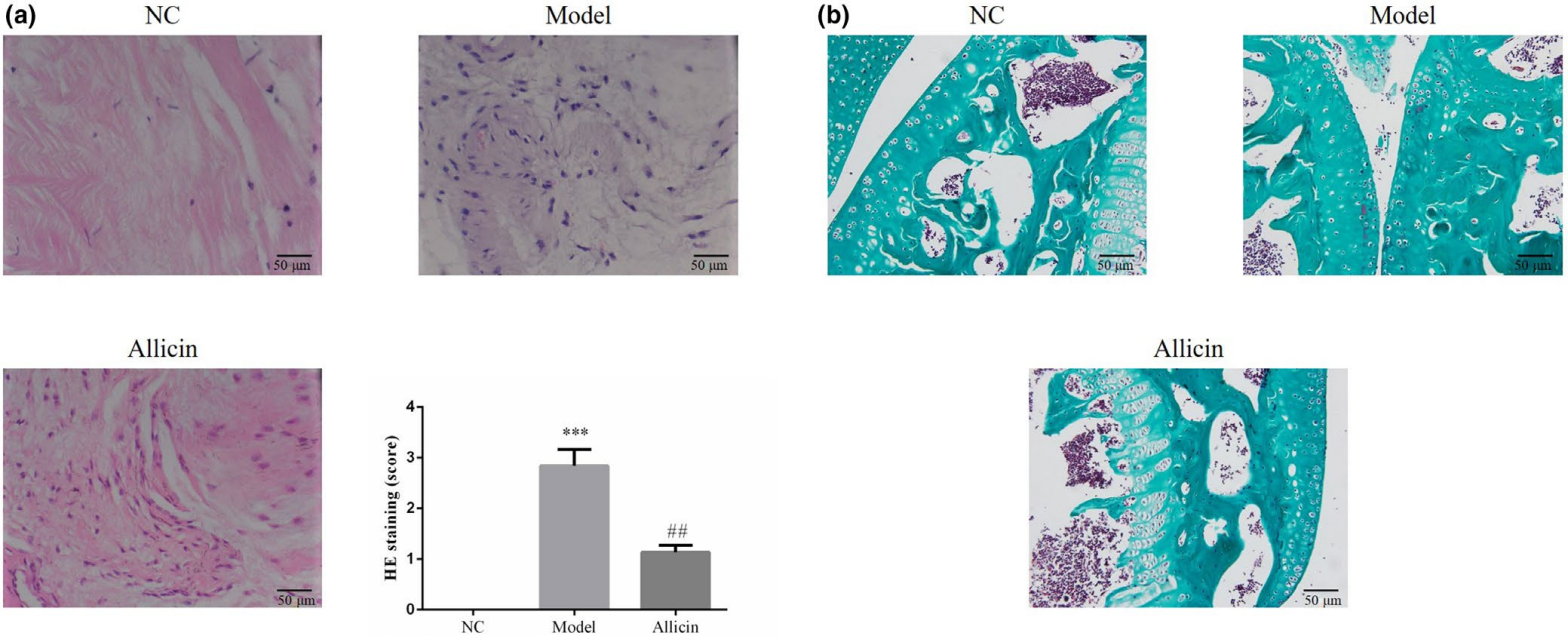
Allicin affects pathological changes of joint in mice. (a) Allicin affects pathological changes of joint in mice by HE staining (200×). ***: *p* < .001, compared with NC group; ##: *p* < .01, compared with Model group. (b) Allicin affects pathological changes of joint in mice by Masson staining (200×)

No significant pathological changes were observed in bone and joint tissues of NC mice after HE staining (Figure 2a). Compared with mice in NC group, mice in Model group had multiple degeneration and necrosis of cartilage surface cells, proliferation of granulation tissue, synovial membrane thickening, disorder of arrangement and extensive erosion to the articular capsule, significant expansion and hyperemia of blood vessels, a large number of inflammatory cell infiltration and osteoclasts (Figure 2a). In Allicin group, there were a small amount of degeneration and necrosis of cartilage surface cells, proliferation of granulation tissue, slight proliferation of synovial tissues, and a small amount of inflammatory cell infiltration around the cartilage surface (Figure 2a). In addition, pathological score results of HE staining showed that the mean pathological score in Model group was significantly higher than that in NC group (*p* < .01); and that in Allicin group was significantly lower than that in Model group (*p* < .01), as shown in Figure 2a. Furthermore, Masson staining showed no significant increase of collagen fibers (Figure 2b) in NC group, large proliferation of collagen fibers and a small amount of elastic fibers in the injured bone and joint (Figure 2b) in Model group, and a small amount of collagen fibers proliferation (Figure 2b) in cartilage surface and bone joint in Allicin group (Figure 2b).

### 3.3 Effect of Allicin on Treg and Th17 ratio of splenic lymphocyte in mice

**FIGURE 3:**
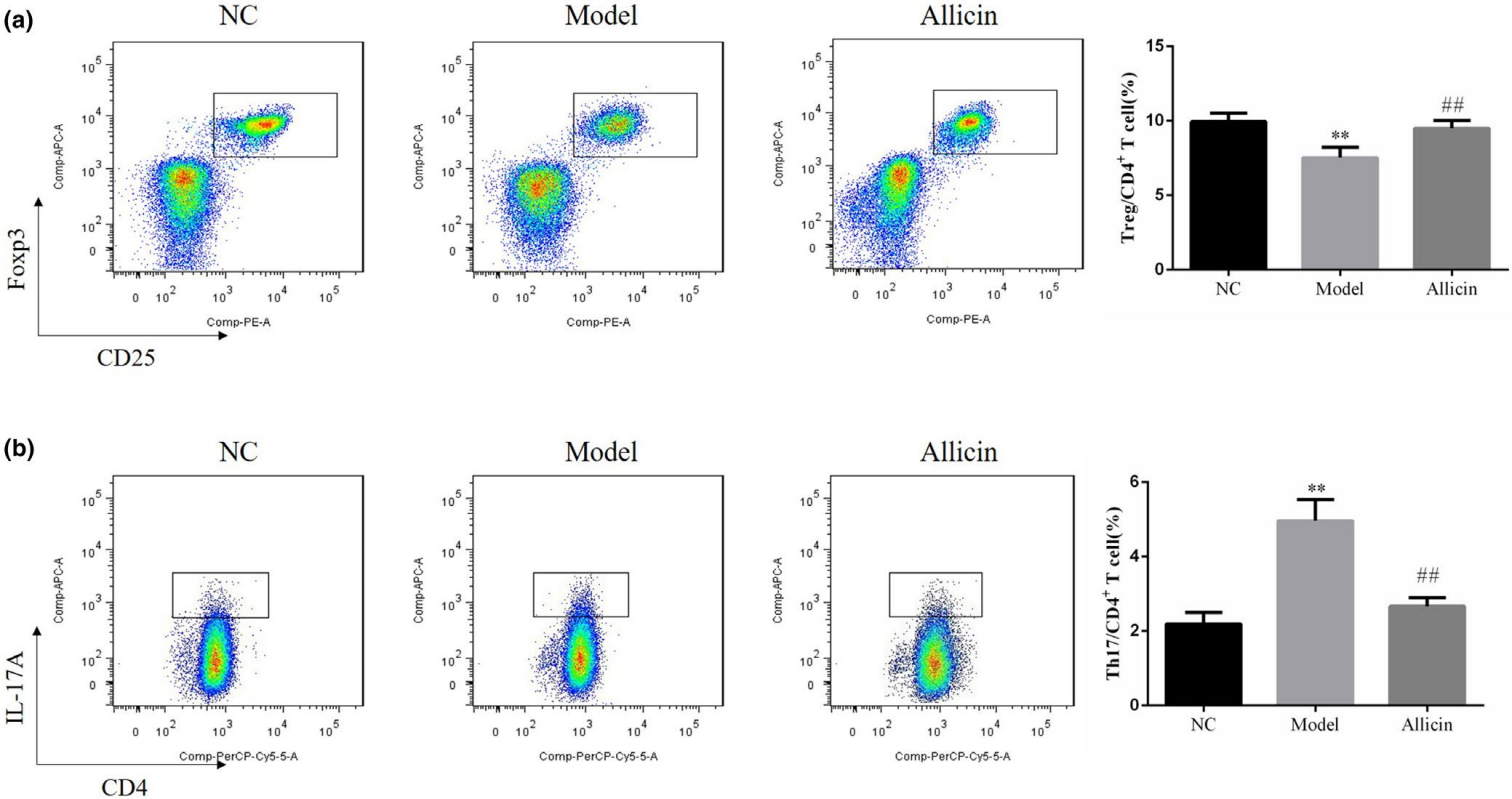
Allicin affects Treg and Th17 in spleen lymphocytes of mice. (a) Allicin affects Treg in spleen lymphocytes of mice. **: *p* < .01, compared with NC group; ##: *p* < .01, compared with Model group. (b) Allicin affects Th17 in spleen lymphocytes of mice. **: *p* < .01, compared with NC group; ##: *p* < .01, compared with Model group

According to the representative chart of the percentage change of cell subsets in splenic lymphocytes, the percentage of Treg cells decreased and the percentage of Th17 increased in splenic lymphocyte of Model group compared with that of NC group, showing statistically significant difference (*p* < .01). Compared with Model group, Treg ratio increased and Th17 ratio decreased in Allicin group, with statistically significant difference (*p* < .01, respectively). It suggested that Allicin can increase Treg and decrease Th17 ratio in splenic lymphocyte (Figure 3).

### 3.4 Effect of Allicin on lymphocyte‐associated proteins in mice

**FIGURE 4:**
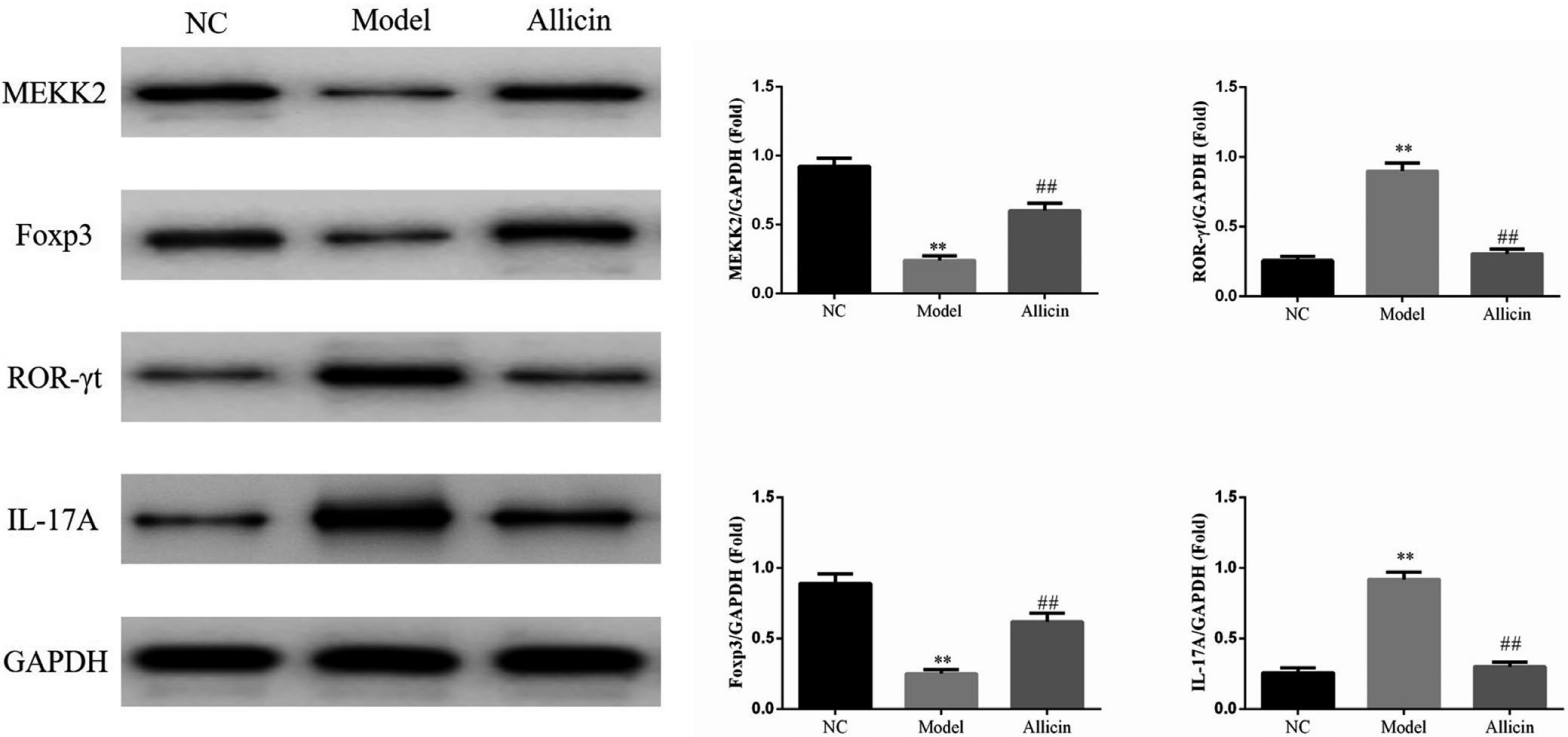
The relative proteins expression by WB assay. **: *p* < .01, compared with NC group; ##: *p* < .01, compared with Model group

Western blotting results showed that the protein expressions of MEKK2 and Foxp3 in Model group were significantly lower than those in NC group (*p* < .01, respectively), while the protein expressions of ROR‐γt and IL‐17A were much higher than those in NC group (*p* < .01, respectively). Compared with Model group, the protein expressions of MEKK2 and Foxp3 protein in Allicin group increased significantly (*p* < .01, respectively), while the protein expressions of ROR‐γt and IL‐17A decreased obviously (*p* < .01, respectively) (Figure 4).

### 3.5 Effect of Allicin on the mRNA expression of Foxp3, ROR‐γt, and IL‐17A

**FIGURE 5:**
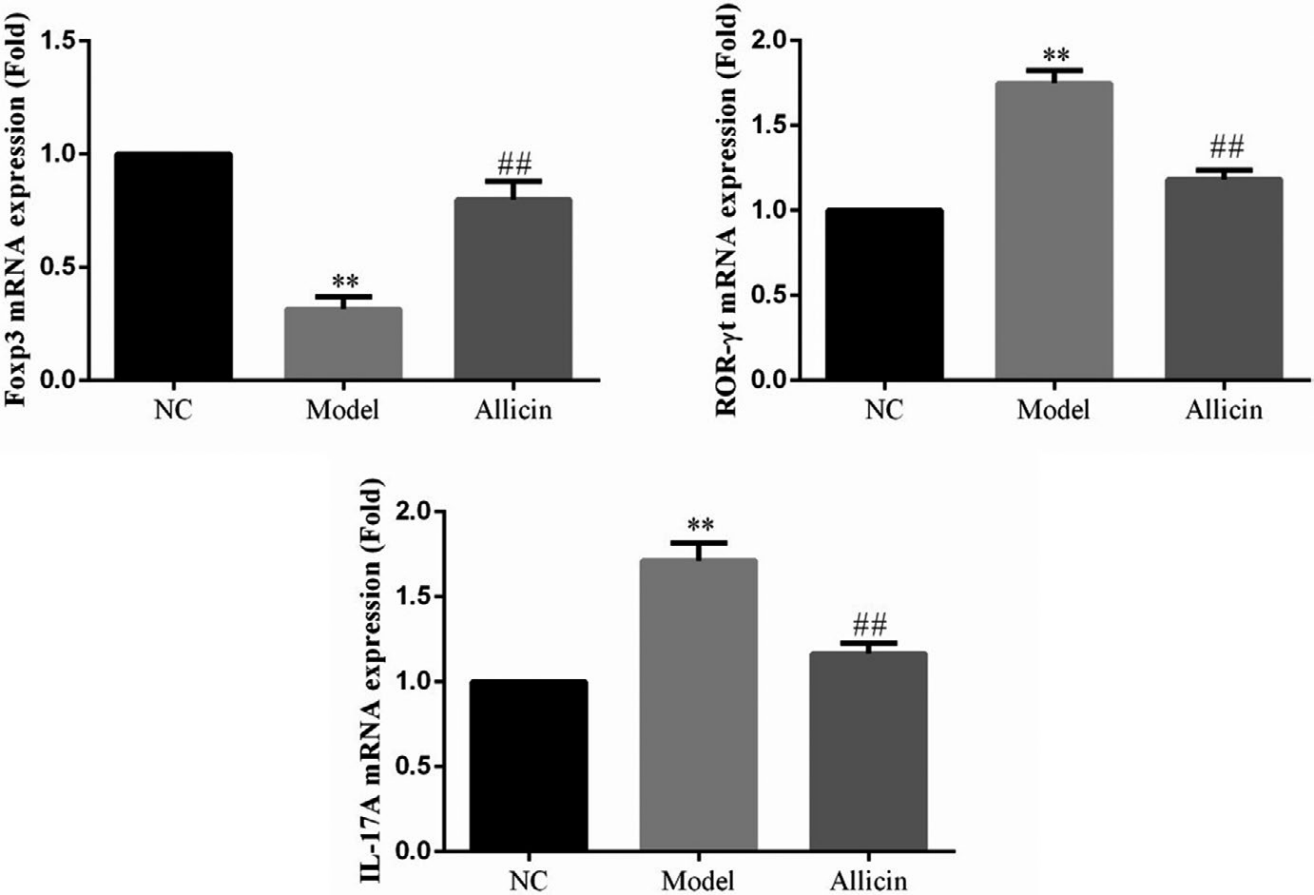
The relative gene expression by RT‐qPCR assay. **: *p* < .01, compared with NC group; ##: *p* < .01, compared with Model group

RT‐qPCR showed that Foxp3 mRNA expression in Model group was obviously lower than that in NC group, while ROR‐γt and IL‐17A mRNA expressions were significantly higher (*p* < .01, respectively). After Allicin treatment, the mRNA expression of Foxp3 was significantly higher than that in Model group, while ROR‐γt and IL‐17A mRNA expressions were significantly lower than those in Model group (*p* < .01, respectively), as shown in Figure 5.

## 4 DISCUSSION

The pathogenesis of RA is still unknown at present. Recent studies have found that abnormal T cell subsets and immune dysfunction play an important role in the pathogenesis of RA. A large number of T cells accumulate in synovial tissue of RA patients, of which T‐cell immunity mediates synovial inflammation and articular cartilage destruction (Aggarwal et al., 2003). CD4^+^T cells are divided into four subgroups: T helper 1 (TH) 1, Th2, Th17, and Treg. Each subgroup regulates mutually and exerts its immunological function together (Zhou et al., 2009).

Th17 and Treg are two subtypes of CD4^+^T cells, and Treg/Th17 imbalance plays an important role in the pathogenesis of RA. Treg is a Th subgroup with immunosuppressive effect. CD4^+^T cells can differentiate into Treg under the action of TGF‐β. It can inhibit the function of T cells by releasing IL‐10 and TEG‐β, and play an immunoregulatory role by reducing the production of inflammatory cytokines and antibody secretion. Treg can inhibit the secretion of IL‐17 by Th17, and then inhibit the autoimmune inflammation induced by IL‐17. Treg/Th 17 balance is an important issue in RA, which may result in disease onset once the balance is destroyed. Therefore, maintaining Th17/Treg balance is of great significance for the treatment of RA (Harrington et al., 2005). Transcription factors play an important role in cell differentiation. Treg‐specific transcription factor forkhead box P3 (Foxp3) is an essential gene controlling the differentiation and function of Treg. Th17 can secrete high levels of characteristic pathogenic cytokine IL‐17A, while nuclear transcription factor retinoic acid‐related lone receptor γt (ROR‐γt) can regulate the production of transcriptional IL‐17 cytokines. The change of ROR‐γt/Foxp3 balance in T cells fundamentally determines the migration of Th17/Treg3 differentiation, and then affects the occurrence and development of RA (Kelly et al., 2017).

MEKKK2 is a member of the MEKK/AET11 subfamily of the MAP3K family in the MAPK signaling pathway, which consists of 619 amino acids. It can phosphorylate the proteins it binds to, mediate the interaction between proteins, and affect their functions (Chayama et al., 2001). MEKKK2 participates in signal regulation of T cells and various cytokines, which can regulate the expression of transcription factors, death receptors and ligands, mediate signal transduction of epidermal growth factor and fibroblast growth factor receptor (Cheng et al., 2005), and induce biological reaction processes such as cell proliferation, differentiation, transformation and apoptosis (Coulombe & Meloche, 2007). Furthermore, CIA is a collagen‐induced inflammatory model of multiple arthritis, which is a classical model of RA. In this animal model, the pathological manifestations of redness and heat pain and related pathogenesis are similar to human RA. It has became a commonly used model at present (Nandakumar & Holmdahl, 2007).

In this study, it was found that Allicin could increase Treg and decrease Th17 ratio in splenic lymphocyte of CIA mice, increase the gene expression of Treg transcription factor Foxp3, decrease the secretion of inflammatory factor IL‐17 of Th17 and transcription factor ROR‐γt in mice. Simultaneously, there were decreased protein and mRNA expression levels of MEKK2 in splenic lymphocytes of CIA model, and the proposed trends could be reversed by Allicin treatment. In addition, Allicin could increase the Treg ratio and decrease the Th17 ratio in splenic lymphocytes of CIA model, thus inhibiting the secretion of inflammatory factor IL‐17A and further suppressing the progress of arthritis.

Chang et al. (2011) reported that MEKK2 and MEKK3 proteins could regulate Th differentiation mediated by TGF‐β. In their research, it was discovered that there was imbalance of Treg and Th17 ratio in peripheral blood of MEKKK2 systematic knockout mice and MEKKK3 conditional knockout mice, which could make EAE mice more susceptible to disease and accumulate more antigen‐specific Th17 in the central nervous system that might be related to the imbalance of T‐cell differentiation, suggesting that the activation of MEK2 and MEKK3 signaling pathways in T cells plays an important regulatory role in inflammation and autoimmune diseases. With respect to the above, the researchers concluded that MEKK2 protein is essential in the pathogenesis of CIA model. Allicin may play a therapeutic role in RA by increasing the expression of MEKK2 protein and affecting Treg/Th17 ratio.

Mitogen‐activated protein kinases are a group of serine/threonine protein kinases which are activated in response to a diverse array of extracellular stimuli. At least four MAPK subfamily members have been found in mammals, including extracellular signal‐regulated protein kinase (ERK) 1/2, c‐Jun N terminal kinase (JNK), p38, and ERK5. Typical activation of MAPK signaling pathway is realized through the cascade reaction of tertiary kinases. Upstream activated MAP3K activates MAP2K and phosphorylates MAPK; and MEKK2 can activate downstream multiple MAPKs (Thalhamer et al., 2008). Kong et al. (2019) found that transient expression of MAP3K2 or MAP3K3 gene in vitro can activate MEKK 2 and MEKK3, and activate downstream proteins including Erk1/2, p38, JNK, and ERK5 at the same time. Meanwhile, the phosphorylated proteins activated by JNK and p38 are highly expressed in synovial cells of synovial tissue in RA (Su et al., 2001), which regulate the production of pathogenic cytokines and are associated with the progression of RA. Besides, previous experiment showed that Allicin could inhibit the proliferation of synovial cells and alleviate synovitis inflammation in RA patients by inhibiting the abnormal activation of RAS‐p38MAPK signaling pathway. In our study, it was speculated that Allicin regulated the downstream four types of MAPK proteins by affecting the expression of MEKK2, thereby affecting Treg/Th17 balance and immune system homeostasis, which, however, remains to be further clarified. It will be our direction of further research to study the changes of downstream signaling pathway of MEKK2 in CIA mice model, and to explore the deep mechanism of MEKK2 regulating Th17/Th17 balance and the pesticide effect of Allicin.

## CONFLICT OF INTEREST

None.

## ETHICAL APPROVAL

This study was approved by Ethics committee of Weifang People's Hospital.

## Data Availability

Research data are not shared.
